# Supplementary material for: Impact of Long-Term Floods on Spatial Dynamics of Myrmica scabrinodis, a Host Ant of a Highly Threatened Scarce Large Blue (Phengaris teleius)
Source: Insects. 2023 Nov 18;14(11):891. doi: 10.3390/insects14110891 (PMC10672202; doi:10.3390/insects14110891)
Supplement: Supplementary file 1 [file insects-14-00891-s001.zip › insects-2683028-supplementary.pdf]

Table S1: List and basic characteristics of variables used in analyses of the impacts of major past floods (years 2010, 2013 and 2017) on the survival rare and spatial recolonization of host ant *Myrmica;* Ljubljansko Barje, Slovenia.

**Variable name**	**Variable description**	**Used as independent / dependent variable**	**Variable type**
ant presence	binary presence of ants in a trap (yes/no)	dependent	binary
number of ants	number of ants in a trap	this continuous variable was used to calculate binary variable "ant presence", but was not included in main analyses	continuous
duration of 2010 flood	duration of 2010 flood on each of the ant trap site (in days)	independent	continuous
duration of 2013 flood	duration of 2013 flood on each of the ant trap sites (in days)	independent	continuous
duration of 2017 flood	duration of 2017 flood on each of the ant trap sites (in days)	independent	continuous
distance to refuge in 2010	distance from the ant trap to the nearest refuge for ants in 2010 flood (in meters)	independent	continuous
distance to refuge in 2013	distance from the ant trap to the nearest refuge for ants in 2013 flood (in meters)	independent	continuous
distance to refuge in 2017	distance from the ant trap to the nearest refuge for ants in 2017 flood (in meters)	independent	continuous
meadow ID	identifier of the meadows (5 ant traps was set in each of the meadows)	independent, used as random factor	attributive
habitat use (in years 2010, 2013, 2017)	use of meadows in given year (3 categories: mowed, cultivated, pasture)	independent	attribute (3 levels)
time elapsed from flood	time elapsed from 2010, 2013 or 2017 flood (in years)	independent	continuous

Table S2: Comparison of competing GLMM models build to analyse effects of past longer floods (in 2010, 2013 and 2017) and distance from the nearest refuge (unflooded area) on the binary presence (YES/NO) of host ant *Myrmica* on Ljubljansko barje, Slovenia. Models were selected based on AICc and build with procedure best subset. Only the models with ΔAICc ≤ 2 are presented.

Competing model	df	AICc	ΔAICc
days flooded 2017 + distance from refuge 2013	2	155,66	
days flooded 2010 + distance from refuge 2013	2	156,34	0,68
days flooded 2013 + distance from refuge 2013	2	156,53	0,87
distance from refuge 2013 + distance from refuge 2017	2	156,92	1,25
distance from refuge 2010 + distance from refuge 2013	2	157,37	1,71
distance from refuge 2013	1	157,66	1,99
days flooded 2013 + distance from refuge 2013 + distance from refuge 2017	3	157,66	2,00
